# Vibration Analysis of Locally Resonant Beams with L-Joint Using an Exact Wave-Based Vibration Approach

**DOI:** 10.3390/ma16062276

**Published:** 2023-03-12

**Authors:** Hangyuan Lv, Rong Zhang, Changji Chen, Hui Ma, Xianzhen Huang, Zhongliang Yu

**Affiliations:** 1 School of Mechanical Engineering and Automation, Northeastern University, Shenyang 110819, China; 2 Key Laboratory of Vibration and Control of Aero Propulsion Systems Ministry of Education of China, Northeastern University, Shenyang 110819, China; 3 China United Network Communication Group Co., Ltd. Liaoning Branch, Shenyang 110027, China; 4 College of New Materials and New Energies, Shenzhen Technology University, Shenzhen 518118, China

**Keywords:** locally resonant beam, low-frequency vibration attenuation, wave-based vibration approach, beam with L-joint

## Abstract

This paper employed and developed the wave-based vibration approach to analyze the band-gap characteristics of a locally resonant (LR) beam with L-joint, which is common in engineering practices. Based on the proposed modular approach, where the discontinuities on the beam are created as modules, the design and modeling work for such an LR beam can be simplified considerably. Then, three kinds of LR beams with an L-joint suspended with transverse-force type resonators and two cells of longitudinal-force-moment type resonators are analyzed, respectively, to show their suppression ability on the axial wave’s propagation and widened effect on the low-frequency band-gaps, where the longitudinal-force-moment type resonators at the 3rd–4th cells can better suppress the propagation of the axial waves. Meanwhile, the proposed analysis results are compared with the ones obtained with the finite element method and further verified the accuracy and efficiency of the wave-based vibration approach. The aim of this paper is to provide an efficient method for the analysis and design of the LR beam with L-joint for low-frequency vibration attenuation in engineering practices.

## 1. Introduction

On account of the perfect characteristics of damping the propagation of flexural waves, the periodic structures of acoustics/elastic metamaterials (AMs/EMs) [1,2,3] generally are applied in flexural wave control. As a kind of metamaterial, phononic crystals (PCs) [4,5] defined as a new functional material are also receiving substantial attention. Furthermore, due to the significant ability of controlling wave propagation [6,7,8], the comprehensive application of periodic structures in engineering correspondingly reflects in the superior damping effect [9,10,11] and efficient energy absorption [12,13], mainly in acoustic and antenna devices.

The band-gap characteristics are considerable owing to the generality of periodic structures, and researchers have begun to intently study various periodic beam structures and further analyze band-gap characteristics [14,15,16,17,18]. Some methods are particularly effective for analyzing quantities of engineering cases. For instance, spectral element method (SEM) [19,20] using higher order polynomials as basis functions, transfer matrix method (TMM) [21,22,23,24] generally applied in the grand partition function, and finite element method (FEM) [25,26,27,28] commonly used in calculation are conventional approaches. In the case of the existing traditional methods, the analysis of modeling and modifying models is still limited. In particular, analyzing the LR beam with L-joint of the common frame in engineering is currently difficult, where relatively advantageous approaches are rarely studied. Therefore, a simple and accurate method needs to be proposed. 

So far, researchers have proposed the exact wave-based vibration approach for analyzing beams and better simplifying the vibration analysis of such structures [29,30,31,32,33,34,35,36,37,38]. Based on the influence of discontinues and planar frame structure, especially on bending and longitudinal vibration, the further interpretation concerning the raised approach is shown by Mei [29,30], which greatly simplifies analysis of the vibration properties of planar frames. In particular, reflection and transmission matrices concerning the beam with L-joint have also been obtained by Mei [31], where the analysis of coupled vibrations in L-shaped and portal planar frame structures is expressed precisely by wave-based vibration approach. In terms of vibrations in built-up multistory space frames, the approach is also provided exactly in Ref. [32], which is extremely accurate in built-up multistory space frames that have mostly been studied numerically. Subsequently, the approach is further extended to analyze vibration properties with a discrete 2DOF spring–mass system in Ref. [33], which is dramatically beneficial to solving vibration problems in combined distributed and discrete systems. Additionally, the analysis of a finite Timoshenko beam carrying force-moment type resonators [34], separated force-moment type resonators [35], and two-degree-of-freedom force type resonators [36] are further studied, where designing and modeling are better simplified by applying the wave-based vibration approach expanded to the band-gap’s analysis, as well as the analysis of lightweight LR beams investigated by Lv et al. [37]. The above studies illustrated the high efficiency of the wave-based vibration approach and made the contribution for further studies. Through incorporating a set of reusable equations as developed by Balaji et al. [38], the above approach is still usable for analyzing the vibration of nonlinear structures. Furthermore, the wave-based vibration approach can relatively simplify vibration-based damage detection methods, similar to making an objective function minimized by an optimization algorithm during an iterative procedure in Refs. [39,40,41]. In general, the proposed method is constantly updated and widely applied by researchers.

In this paper, the band-gap characteristics of the LR beam with L-joint is further analyzed through the developed wave-based method. Based on the proposed approach, the suspended resonators are considered as forces and moments that the host beam receives incident waves. Then, the propagation, reflection, and transmission matrices at discontinuities (boundaries, resonators attached points, L-joint, etc.) on the host beam can be created as modules with MATLAB software. The design and modeling task of such an LR beam can be built and modified easily through combining the specific modules. Comparing the analysis result with FEM, the accuracy and efficiency of the proposed wave-based analysis approach has been verified as being better by Leamy [42]. The differences of FRF curves and band-gaps are obviously shown in Section 4. Moreover, two other types of LR beams with longitudinal-force-moment type resonators are further analyzed to discuss their widened effect on the band-gaps of the structure. When the longitudinal-force-moment type resonators suspended at the 3rd–4th cells of the LR beam with L-joint, the suppression of the axial wave’s propagation at low-frequency range is better. In this paper, an effective means for design and analysis of the LR beam with L-joint is elucidated, which is common in engineering practices such as studying band-gaps’ behavior, propagation characteristics, and dispersion properties, especially for vibration attenuation such as in the framework and space-arm of antennas. Other methods were not chosen mainly because the better accuracy, good applicability, and repeatability of procedures of the wave-propagation method are advantageous for analyzing LR beams with L-joint.

### Notation

The parameters and variables in this paper are listed in Table 1, and Table 2 summarizes the key abbreviations commonly used in this paper.

## 2. Wave-Based Analysis Methodology

### 2.1. Overview

The kinematic equations in Ref. [43] of Timoshenko beams concerning bending, rotation, and longitudinal vibration can be represented as follows:

(1) $$GA\kappa \left(\frac{\partial \psi \left(x,t\right)}{\partial x}-\frac{\partial^{2}w\left(x,t\right)}{\partial x^{2}}\right)+\rho A\frac{\partial^{2}w\left(x,t\right)}{\partial t^{2}}=q\left(x,t\right)$$

(2) $$EI\frac{\partial^{2}\psi \left(x,t\right)}{\partial x^{2}}+GA\kappa \left(\frac{\partial w\left(x,t\right)}{\partial x}-\psi \left(x,t\right)\right)-\rho I\frac{\partial^{2}\psi \left(x,t\right)}{\partial t^{2}}=0$$

(3) $$\rho A\frac{\partial^{2}u\left(x,t\right)}{\partial t^{2}}-EA\frac{\partial^{2}u\left(x,t\right)}{\partial x^{2}}=p\left(x,t\right)$$

where t is time, ψ(x,t) is the rotation angle of the entire flexing section, x is the neutral line position along the beam, and $\partial w\left(x,t\right)/\partial x$ represents the gradient of beam centerline; the transverse and longitudinal deflection are denoted as w(x,t) and u(x,t), respectively. q(x,t) and p(x,t) are signified as the longitudinal and transverse force applied on each unit length. The shear modulus G, mass density ρ, and Young’s modulus E are the material parameters. The geometric parameters include shear coefficient κ, area moment of inertia I, and area of the cross section A. 

The kinematic variables relating to w(x,t), ψ(x,t), and u(x,t) can be, respectively, calculated as Equations (4)–(6), where V(x,t) is the shear force, the bending moment is symbolized as M(x,t), and longitudinal force is denoted as F(x,t).

(4) $$V(x,t)=GA\kappa \left(\frac{\partial w(x,t)}{\partial x}-\psi (x,t)\right)$$

(5) $$M(x,t)=EI\frac{\partial \psi (x,t)}{\partial x}$$

(6) $$F(x,t)=EA\frac{\partial u(x,t)}{\partial x}$$

The free-wave propagation Equations (1)–(3) can be calculated as Equations (7)–(9) due to few considerations concerning loading and $e^{i\omega t}$ signified as the restraint time dependence.

(7) $$w\left(x,t\right)=a_{1}^{+}e^{-ik_{1}x}+a_{2}^{+}e^{{-k}_{2}x}+a_{1}^{-}e^{ik_{1}x}+a_{2}^{-}e^{k_{2}x}$$

(8) $$\psi \left(x,t\right)=-iPa_{1}^{+}e^{-ik_{1}x}-Na_{2}^{+}e^{-k_{2}x}+iPa_{1}^{-}e^{ik_{1}x}+Na_{2}^{-}e^{k_{2}x}$$

(9) $$u\left(x,t\right)=c^{+}e^{-ik_{3}x}+c^{-}e^{ik_{3}x}$$

where a superscript + or − signifies the forward or backward propagating waves; $k_{1},k_{2},k_{3}$ are envisaged as three wavenumbers; $a_{1}$, $a_{2}$ and c correspondingly indicate the components of the propagating waves from one end of the beam, which can be found in the cited reference [42]. The correlation of both the rotational solution and the transverse displacement solution is through iP and N given by:

(10) $$P=k_{1}\left(1-\frac{\omega^{2}}{k_{1}^{2}C_{s}^{2}}\right),\;\;\;N=k_{2}\left(1+\frac{\omega^{2}}{k_{2}^{2}C_{s}^{2}}\right)$$

The above relations concerning the wavenumber-frequency dispersion are as follows:

(11) $$k_{1}=\sqrt{\frac{1}{2} [{\left(\frac{1}{C_{s}}\right)}^{2}+{\left(\frac{C_{r}}{C_{b}}\right)}^{2}]\omega^{2}+\sqrt{{{\left(\frac{\omega }{C_{b}}\right)}^{2}+\frac{1}{4}[{\left(\frac{1}{C_{s}}\right)}^{2}-{\left(\frac{C_{r}}{C_{b}}\right)}^{2}]}^{2}\omega^{4}}}$$

(12) $$k_{2}=\sqrt{-\frac{1}{2} [{\left(\frac{1}{C_{s}}\right)}^{2}+{\left(\frac{C_{r}}{C_{b}}\right)}^{2}]\omega^{2}+\sqrt{{{\left(\frac{\omega }{C_{b}}\right)}^{2}+\frac{1}{4}[{\left(\frac{1}{C_{s}}\right)}^{2}-{\left(\frac{C_{r}}{C_{b}}\right)}^{2}]}^{2}\omega^{4}}}$$

(13) $$k_{3}=\sqrt{\frac{E}{\rho }\omega^{2}}$$

where the coefficients of rotation, bending, and shear wave speeds are correspondingly shown as follows:

(14) $$C_{r}=\sqrt{\frac{\rho I}{\rho A},\;\;}C_{b}=\sqrt{\frac{EI}{\rho A}},\;\;C_{s}=\sqrt{\frac{GA\kappa }{\rho A}}$$

### 2.2. Propagation Matrix

**Figure 1:**
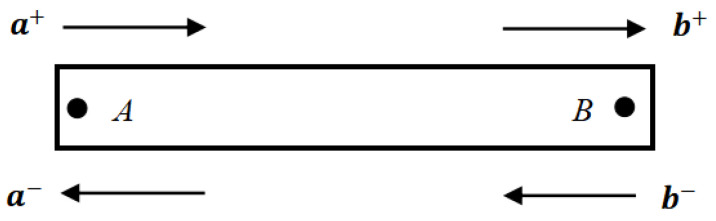
Wave propagation between two points of a uniform beam at a distance of x. (Reprinted from Ref. [37].)

With the existence of discontinuities along a uniform beam, the propagation situation of the exact wave is expressed by Equations (7)–(9) with a single frequency, assuming the distance from point *A* to *B* in a beam is x as represented in Figure 1 [37]. The relations of propagation matrix $f\left(x\right)$ and wave vectors at *A* and *B* are defined as:

(15) $$b^{+}=f\left(x\right)a^{+},a^{-}=f(x)b^{-}$$

in which

(16) $$a^{+}= [\begin{array}{c} a_{1}^{+}\\ a_{2}^{+}\\ c^{+} \end{array}],a^{-}= [\begin{array}{c} a_{1}^{-}\\ a_{2}^{-}\\ c^{-} \end{array}],b^{+}= [\begin{array}{c} b_{1}^{+}\\ b_{2}^{+}\\ d^{+} \end{array}],b^{-}= [\begin{array}{c} b_{1}^{-}\\ b_{2}^{-}\\ d^{-} \end{array}]$$

Where $a^{+}$, $b^{+}$, $a^{-}$**,** and $b^{-}$**,** respectively, signify the wave coefficients of forward propagation and opposite propagation at points *A* and *B*. Specifically, $a_{1}^{\pm }$, $a_{2}^{\pm }$, $b_{1}^{\pm }$, $b_{2}^{\pm }$ indicate the components of bending wave, and $c^{\pm }$, $d^{\pm }$ denote longitudinal wave components. These wave modes can be found in the cited reference [31].

(17) $$f\left(x\right)= [\begin{array}{ccc} e^{-ik_{1}x} & 0 & 0\\ 0 & e^{-k_{2}x} & 0\\ 0 & 0 & e^{-{ik}_{3}x} \end{array}]$$

### 2.3. Reflection at a Free Boundary

The transverse forces and bending moments are considered few in this paper when the LR beam is in the situation of free endless boundary. The connection between $a^{-}$ and $a^{+}$**,** which are the counterpart to the reflected and incident waves, is shown as (18), and a reflection matrix can be established as (19).

(18) $$a^{-}=r_{f}a^{+}$$

where

(19) $$r_{f}= [\begin{array}{ccc} \frac{-Pk_{1}\left(-N+k_{2}\right)+ik_{2}N(k_{1}-P)}{Pk_{1}\left(-N+k_{2}\right)+ik_{2}N(k_{1}-P)} & \frac{2Nk_{2}(-N+k_{2})}{Pk_{1}\left(-N+k_{2}\right)+ik_{2}N(k_{1}-P)} & 0\\ \frac{2iPk_{1}(-P+k_{1})}{Pk_{1}\left(-N+k_{2}\right)+ik_{2}N(k_{1}-P)} & \frac{Pk_{1}\left(-N+k_{2}\right)-ik_{2}N(k_{1}-P)}{Pk_{1}\left(-N+k_{2}\right)+ik_{2}N(k_{1}-P)} & 0\\ 0 & 0 & 1 \end{array}]$$

### 2.4. Applied Forces and Moments

**Figure 2:**
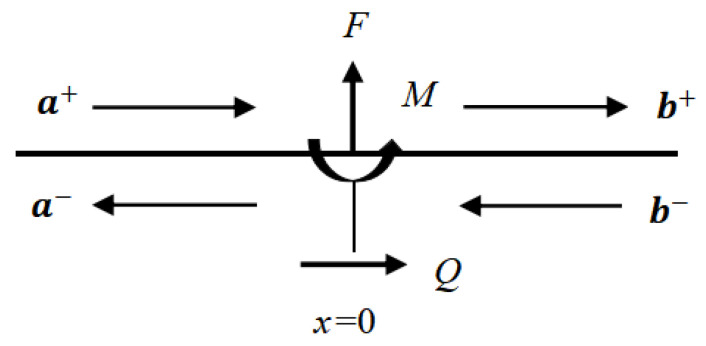
Waves generated by external force. (Reprinted from Ref. [37].).

At the point *x* = 0, the waves a and b are formed by the external forces and moment as depicted in Figure 2 [37], where transverse forces, longitudinal forces, bending moments are defined as *F*, *Q*, and *M*, and the equilibrium and continuity equations are:

(20) $$b^{+}-a^{+}=f+m-q$$

(21) $$b^{-}-a^{-}=-f+m+q$$

where the amplitude vector of the excited wave is given:

(22) $$f= [\begin{array}{c} iN\\ P\\ 0 \end{array}]\frac{F}{GA\kappa (k_{2}P-k_{1}N)}$$

(23) $$m= [\begin{array}{c} 1\\ -1\\ 0 \end{array}]\frac{M}{EI(k_{1}P+k_{2}N)}$$

(24) $$q= [\begin{array}{c} 0\\ 0\\ i \end{array}]\frac{Q}{EAk_{3}}$$

### 2.5. Transmission and Reflection at L-Joint

**Figure 3:**
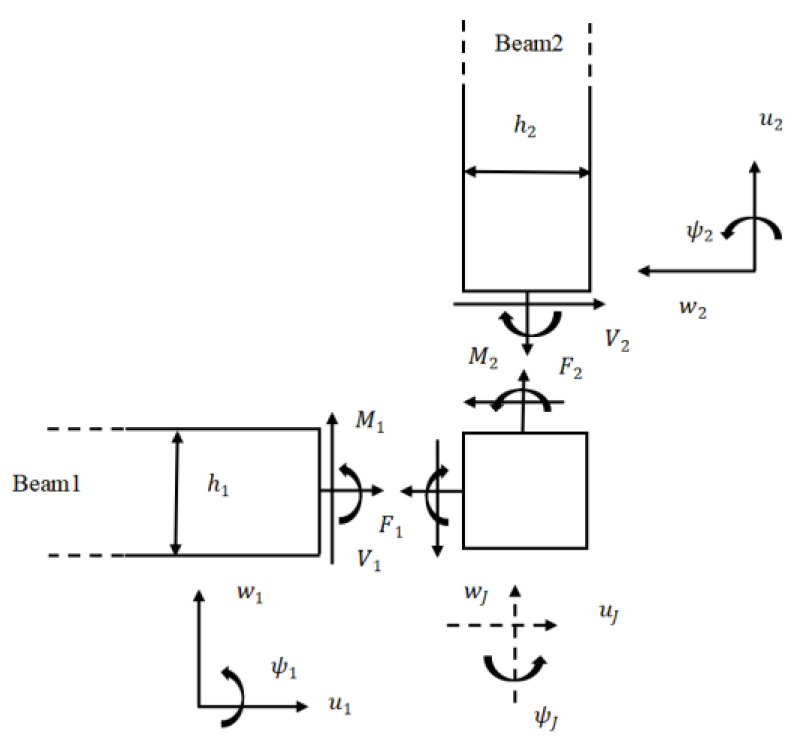
Free body diagram of an L-joint. (Reprinted with permission from Ref. [31]. 2012, ASME Publishing.).

At the L-joint, reflected and transmitted bending and axial waves are generated at the members attached to the joint from an injected bending wave. Evidently, based on the coupled equilibrium and continuity relations, the free body diagram analysis of the L-joint in planar motion is shown in Figure 3 [31]. The equations of motion for the L-joint in Figure 3 are denoted in Equations (25)–(27), where *F* and *V* are the axial and transverse force, and the beam thickness is *h.* The joint displacements are $u_{J}$, $w_{J}$, and rotation is $\psi_{J}$.

The first two of these equations include the mass of the L-joint, where the third includes the moment of inertia of the joint and the moments generated by the off-set forces.

(25) $$F_{2}-V_{1}=m{w_{J}}^{\prime \prime }$$

(26) $$-V_{2}-F_{1}=m{u_{J}}^{\prime \prime }$$

(27) $$M_{2}-M_{1}+V_{1}\frac{h_{2}}{2}+V_{2}\frac{h_{1}}{2}=J{\psi_{J}}^{\prime \prime }$$

The continuity equations at the joint are:

(28) $$u_{1}=u_{2}=u_{J}$$

(29) $$w_{1}=w_{J}-\frac{h_{2}}{2}\psi_{J,}w_{2}=-u_{J}+\frac{h_{1}}{2}\psi_{J}$$

(30) $$\psi_{1}=\psi_{2}=\psi_{J}$$

The axial force, bending moment, and shear force are below:

(31) $$F_{1}={\left(EA\right)}_{1}\frac{\partial u_{1}}{\partial x_{1}},F_{2}={\left(EA\right)}_{2}\frac{\partial u_{2}}{\partial x_{2}}$$

(32) $$M_{1}={\left(EI\right)}_{1}\frac{\partial \psi_{1}}{\partial x_{1}},M_{2}={\left(EI\right)}_{2}\frac{\partial \psi_{2}}{\partial x_{2}}$$

(33) $$V_{1}={\left(GA\kappa \right)}_{1}\left(\frac{\partial w_{1}}{\partial x_{1}}-\psi_{1}\right),V_{R}={\left(GA\kappa \right)}_{2}\left(\frac{\partial w_{2}}{\partial x_{2}}-\psi_{2}\right)$$

where the distance along each beam axis is represented x.

**Figure 4:**
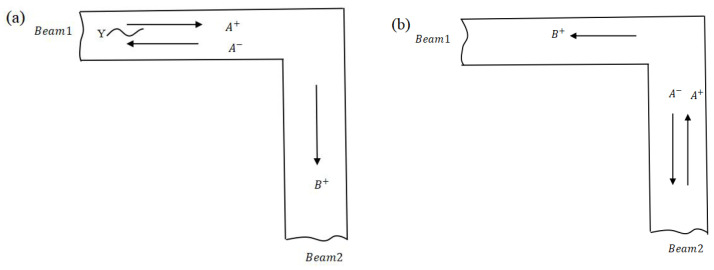
Wave reflection and transmission at an L-joint, (**a**) waves incident from beam 1, (**b**) waves incident from beam 2. (Reprinted with permission from Ref. [31]. 2012, ASME Publishing.).

In Figure 4 [31], a set of positive incident waves $A^{+}$ upon the L-joint from one beam produces opposite and transmitted waves $A^{-}$ and $B^{+}$, where the relationship about $A^{+}$ between the matrices r and t of reflection and transmission are as follows:

***A^−^*** = ***r* *A^+^***, ***B^+^*** = ***t* *A^+^*** (34)

where

(35) $$A^{+}= [\begin{array}{c} a_{1}^{+}\\ a_{2}^{+}\\ c^{+} \end{array}],A^{-}= [\begin{array}{c} a_{1}^{-}\\ a_{2}^{-}\\ c^{-} \end{array}],B^{+}= [\begin{array}{c} b_{1}^{+}\\ b_{2}^{+}\\ d^{+} \end{array}]$$

In Equation (35), ${a_{1}}^{+}$, ${a_{1}}^{-}$, ${b_{1}}^{+}$, respectively, denote the incident, reflected, and transmitted propagating bending waves; ${a_{2}}^{+}$, ${a_{2}}^{-}$, ${b_{2}}^{+}$ correspondingly show the incident, reflected, and transmitted near-field bending waves; and the incident, reflected, and transmitted axial propagating waves are represented as $c^{+}$, $c^{-}$, $d^{+}$, respectively [31]. For axial waves of the frequency up to twice the cut-off frequency of Timoshenko bending waves, the elementary, one-dimensional theory is especially adopted.

Firstly, the incident wave is considered from beam 1. Assuming that beams 1 and 2 have the same material properties to simplify our analysis, one has:

(36) $$w_{1}=a_{1}^{+}e^{-ik_{1}x_{1}}+a_{2}^{+}e^{{-k}_{2}x_{1}}+a_{1}^{-}e^{ik_{1}x_{1}}+a_{2}^{-}e^{k_{2}x_{1}}$$

(37) $$u_{1}=c^{+}e^{-ik_{3}x_{1}}+c^{-}e^{ik_{3}x_{1}}$$

(38) $$\psi_{1}=-iPa_{1}^{+}e^{-ik_{1}x_{1}}-Na_{2}^{+}e^{-k_{2}x_{1}}+iPa_{1}^{-}e^{ik_{1}x_{1}}+Na_{2}^{-}e^{k_{2}x_{1}}$$

(39) $$w_{2}=b^{+}e^{-ik_{1}x_{2}}+{b_{N}}^{+}e^{{-k}_{2}x_{2}}$$

(40) $$u_{2}=d^{+}e^{-ik_{3}x_{2}}$$

(41) $$\psi_{2}=-iPb^{+}e^{-ik_{1}x_{2}}-N{b_{N}}^{+}e^{-k_{2}x_{2}}$$

The transmission and reflection matrices $t_{12}$, $r_{11}$ and $t_{21}$, $r_{22}$ can be derived from solving Equations (25)–(34) and (36)–(41), respectively, corresponding to an incident wave from beam 1 and beam 2. These specific expressions are not represented here but can be found in the cited reference [31]. 

### 2.6. Transmission and Reflection at the Transverse-Force Type Resonator Attached Point

**Figure 5:**
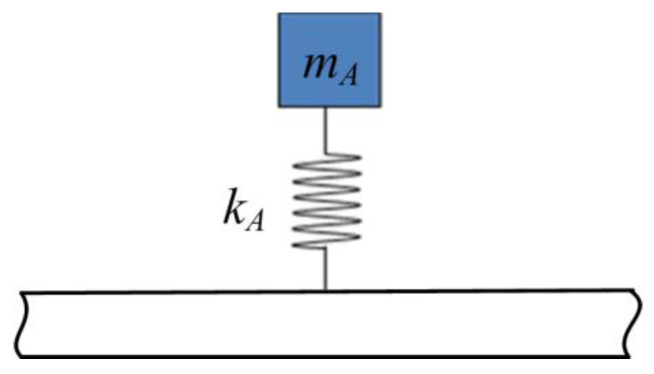
A host beam with a transverse-force type resonator. (Reprinted from Ref. [34].).

**Figure 6:**
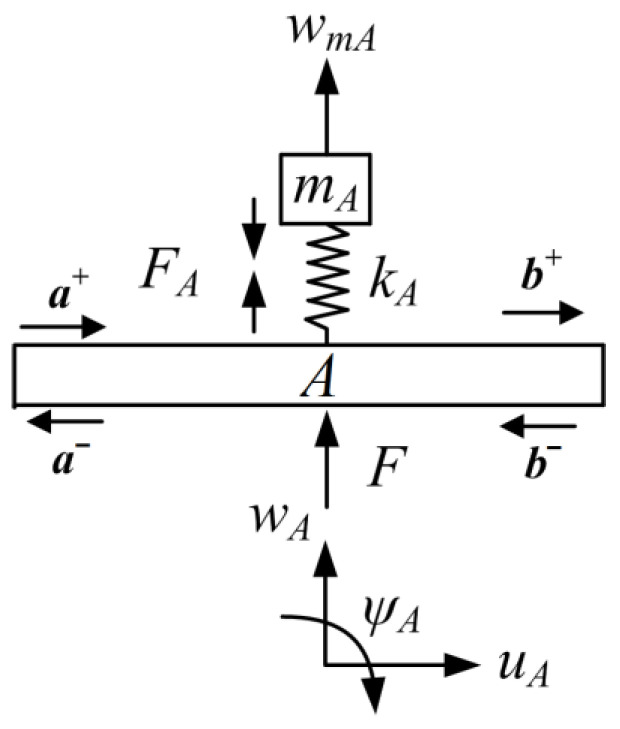
Free body diagram of a transverse-force type resonator. (Reprinted from Ref. [34].).

The transverse-force type resonator is shown in Figure 5 [34]. We name this type of resonator as transverse-force type because the one applies transverse force to the host beam, as depicted in Figure 6 [34]. In the figure, $m_{A}$ is the mass of the resonator and $k_{A}$ is the stiffness of the linear elastic spring. $w_{mA}$ is the transverse deflection of the mass blocks $m_{A}$. The spring induces the force $F_{A}$, and *F* is the force generated from the resonator and applied on the host beam. At point *A* of the major beam, $w_{A}$, $u_{A}$, and $\psi_{A}$ are, respectively, the deflections of transverse, axial, and angular rotation. Here, the axial deflection $u_{A}$ and angular rotation $\psi_{A}$ are considered few because of no application to the host beam of the axial force and bending moment by the transverse-force type resonator. 

According to Figure 6, the motion expression of the resonator is:

(42) $$-F_{A}=m{\ddot{w}}_{mA}$$

where

(43) $$F_{A}=k_{A}(w_{mA}-w_{A})$$

Assuming that the total motion is time harmonic with frequency ω, through the conjunction of Equations (42) and (43), the displacements at point *A* of the mass block $w_{mA}$ correlating of the deflection $w_{A}$ is expressed as:

(44) $$w_{mA}=\frac{k_{A}}{k_{A}-m_{A}\omega^{2}}w_{A}$$

Then, the force F can be expressed by combining Equations (43) and (44):

(45) $$F=F_{A}=r_{A}\cdot w_{A}$$

where

$$r_{A}=\frac{m_{A}k_{A}\omega^{2}}{k_{A}-m_{A}\omega^{2}}$$

As states in Ref. [37], the production of applied force by the resonators is considered, where the host beam receives injecting waves. Combining Equations (7)–(9), (20) and (21), and uniting the expressions of transverse force F in Equation (45) with Equations (22)–(24), the relations of vibration waves at point *A* can be obtained as:

(46) $$[\begin{array}{cc} A_{p1} & A_{n1}\\ A_{p2} & A_{n2} \end{array}] [\begin{array}{c} a^{+}\\ a^{-} \end{array}]+ [\begin{array}{cc} B_{p1} & B_{n1}\\ B_{p2} & B_{n2} \end{array}] [\begin{array}{c} b^{+}\\ b^{-} \end{array}]=0$$

where the coefficient matrices in Equation (35) are:

$$A_{p1}= [\begin{array}{ccc} ir_{A}N\beta_{1}+1 & ir_{A}N\beta_{1} & 0\\ r_{A}P\beta_{1} & -r_{A}P\beta_{1} & 0\\ 0 & 0 & -1 \end{array}]\;A_{p2}= [\begin{array}{ccc} -ir_{A}N\beta_{1} & -ir_{A}N\beta_{1} & 0\\ -r_{A}P\beta_{1} & -r_{A}P\beta_{1} & 0\\ 0 & 0 & 0 \end{array}]\;A_{n1}= [\begin{array}{ccc} ir_{A}N\beta_{1} & ir_{A}N\beta_{1} & 0\\ r_{A}P\beta_{1} & r_{A}P\beta_{1} & 0\\ 0 & 0 & 0 \end{array}]\;A_{n2}= [\begin{array}{ccc} -ir_{A}N\beta_{1}+1 & -ir_{A}N\beta_{1} & 0\\ -r_{A}P\beta_{1} & -r_{A}P\beta_{1}+1 & 0\\ 0 & 0 & -1 \end{array}]\;B_{p1}=B_{n2}= [\begin{array}{ccc} -1 & 0 & 0\\ 0 & -1 & 0\\ 0 & 0 & -1 \end{array}]\;B_{p2}=B_{n1}= [\begin{array}{ccc} 0 & 0 & 0\\ 0 & 0 & 0\\ 0 & 0 & 0 \end{array}]\;\beta_{1}=\frac{1}{2(GA\kappa )(k_{2}P-k_{1}N)}$$

### 2.7. Transmission and Reflection at the Longitudinal Force-Moment Type Resonator Attached Point

**Figure 7:**
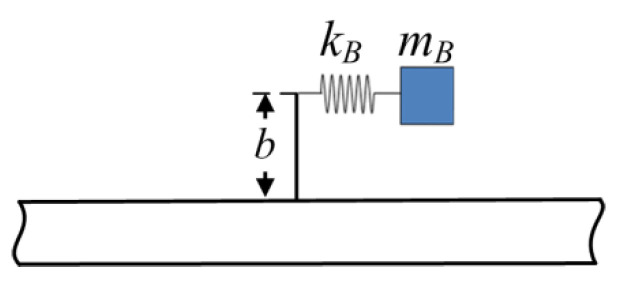
A host beam with a longitudinal-force-moment type resonator.

Figure 7 depicts the longitudinal-force-moment type resonator; this type of resonator applies both longitudinal force and bending moment to the host beam. In the figure, $m_{B}$ is the mass of the resonator and $k_{B}$ is the stiffness of the linear elastic spring. 

**Figure 8:**
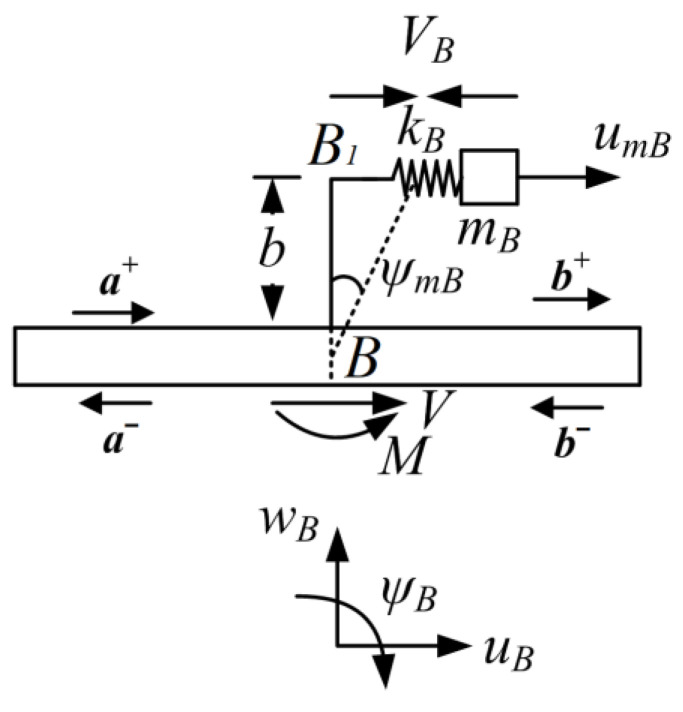
Free body diagram of a longitudinal force-moment type resonator.

The free body diagram of the resonator is shown in Figure 8. In the figure, $u_{mB}$ and $\psi_{mB}$ are the axial deflections of the mass blocks $m_{B}$ and angular rotation of the beam *BB*_1_, respectively. $V_{B}$ is the longitudinal force caused by the spring. The production of longitudinal force *V* and bending moment *M* are generated from the resonator, which are applied on the host beam, respectively. At point *B* on the beam, the deflection of transverse, axial and angular rotation are $w_{A}$, $u_{A}$, and $\psi_{A}$, respectively. Note that the transverse $w_{A}$ is not involved because of no generation of transverse force of this type of resonator to the host beam. 

As shown in Figure 8, the motion expression of the resonator is:

(47) $$-V_{B}=m_{B}{\ddot{u}}_{mB}$$

where

(48) $$V_{B}=k_{B}(u_{mB}-u_{B}-\psi_{B}\cdot b)$$

(49) $$M=V_{B}\cdot b$$

Similarly, considering the subjection of time harmonic with frequency ω to the total motion, through uniting Equations (47) and (48), the displacements correlation of the mass block $u_{mB}$ and the deflection $u_{B}$ at the attachment point is shown as:

(50) $$u_{mB}=\frac{k_{B}}{k_{B}-m_{B}\omega^{2}}{(u}_{B}+b\cdot \psi_{B})$$

Then, the force V can be expressed by combining Equations (47)–(50):

(51) $$V=V_{B}=r_{B}\cdot {(u}_{B}+b\cdot \psi_{B})$$

(52) $$M_{B}=r_{B}\cdot {b\cdot (u}_{B}+b\cdot \psi_{B})$$

where

$$r_{B}=\frac{m_{B}k_{B}\omega^{2}}{k_{B}-m_{B}\omega^{2}}$$

Combining Equations (7)–(9), (20) and (21), and uniting the expressions of transverse force V and bending moment *M* in Equations (51) and (52) with Equations (22)–(24), the relations of vibration waves at point *B* can be obtained as:

(53) $$[\begin{array}{cc} C_{p1} & C_{n1}\\ C_{p2} & C_{n2} \end{array}] [\begin{array}{c} a^{+}\\ a^{-} \end{array}]+ [\begin{array}{cc} D_{p1} & D_{n1}\\ D_{p2} & D_{n2} \end{array}] [\begin{array}{c} b^{+}\\ b^{-} \end{array}]=0$$

where the coefficient matrices in Equation (53) are:

$$C_{p1}= [\begin{array}{ccc} -ir_{B}P\beta_{2}b^{2}+1 & -r_{B}N\beta_{2}b^{2} & r_{B}\beta_{2}b\\ ir_{B}P\beta_{2}b^{2} & r_{B}N\beta_{2}b^{2}+1 & -r_{B}\beta_{2}b\\ r_{B}P\beta_{3}b & {ir}_{B}N\beta_{3}b & -{ir}_{B}\beta_{3}+1 \end{array}]\;C_{p2}= [\begin{array}{ccc} -ir_{B}P\beta_{2}b^{2} & -r_{B}N\beta_{2}b^{2} & r_{B}\beta_{2}b\\ ir_{B}P\beta_{2}b^{2} & r_{B}N\beta_{2}b^{2} & -r_{B}\beta_{2}b\\ r_{B}P\beta_{3}b & -{ir}_{B}N\beta_{3}b & {ir}_{B}\beta_{3} \end{array}]\;C_{n1}= [\begin{array}{ccc} ir_{B}P\beta_{2}b^{2} & r_{B}N\beta_{2}b^{2} & r_{B}\beta_{2}b\\ -ir_{B}P\beta_{2}b^{2} & {-r}_{B}N\beta_{2}b^{2} & -r_{B}\beta_{2}b\\ r_{B}P\beta_{3}b & -{ir}_{B}N\beta_{3}b & -{ir}_{B}\beta_{3} \end{array}]\;C_{n2}= [\begin{array}{ccc} ir_{B}P\beta_{2}b^{2}+1 & r_{B}N\beta_{2}b^{2} & r_{B}\beta_{2}b\\ -ir_{B}P\beta_{2}b^{2} & {-r}_{B}N\beta_{2}b^{2}+1 & -r_{B}\beta_{2}b\\ {-r}_{B}P\beta_{3}b & {ir}_{B}N\beta_{3}b & {ir}_{B}\beta_{3}+1 \end{array}]\;D_{p1}=D_{n2}= [\begin{array}{ccc} -1 & 0 & 0\\ 0 & -1 & 0\\ 0 & 0 & -1 \end{array}]\;D_{p2}=D_{n1}= [\begin{array}{ccc} 0 & 0 & 0\\ 0 & 0 & 0\\ 0 & 0 & 0 \end{array}]\;\beta_{2}=\frac{1}{2(EI)(k_{1}P+k_{2}N)}\;\beta_{3}=\frac{1}{2EAk_{3}}$$

## 3. Vibration Analysis with Wave-based Approach

**Figure 9:**
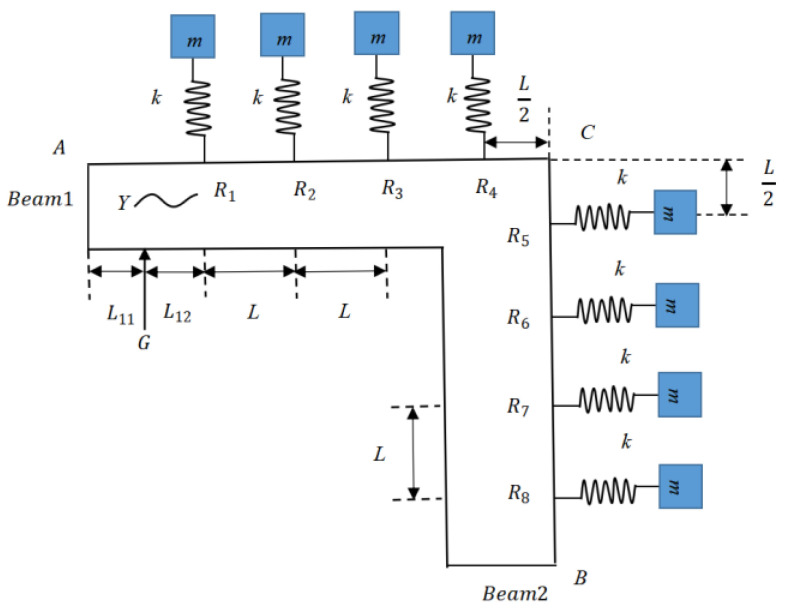
Equivalent model of the proposed LR beam for the wave-based approach.

In this section, we apply the related theory in Section 2 to the vibration analysis of the LR beam with L-joint. The first exhibition of the studied LR beam is in Figure 9. In the figure, eight transverse-force type resonators are suspended on the host beam, where the mass of the resonator is signified as m, the stiffness of the linear elastic spring is depicted as k, and L is the distance from the previous resonator to the next of the LR beam. Exerting the additional force at point *G*, the corresponding distances of the left and right at point *G* are $L_{11}$ and $L_{12}$. In addition, the distances both of the left in the horizontal direction and the vertical direction at point *C* are L/2.

**Figure 10:**
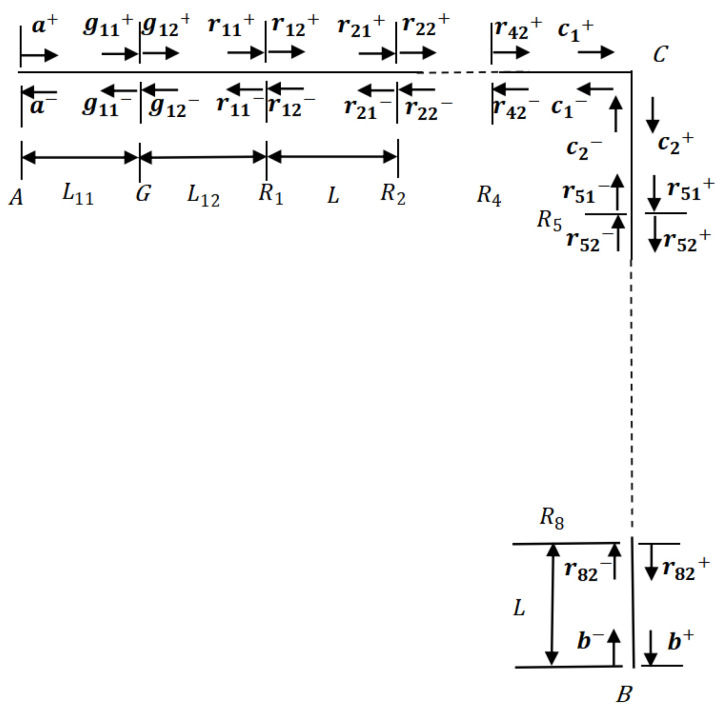
Vibration wave components in the LR beam.

Figure 10 illustrates the vibration wave components in the LR beam. As shown in the figure, the transverse-force resonators are attached at points *R*_1_, *R*_2_, *R*_3_, *R*_4_, *R*_5_, *R*_6_, *R*_7_, and *R*_8_. Based on the free experimental measurements solution adopted by Yu et al. [44], when the band-gap is wide enough under free–free boundary condition, the detailed analysis process is relatively easier. Thus, the boundary condition of the LR beam at *A* and *B* is free. At point *G*, the additional force is exerted, causing the waves $g_{11}^{+},g_{11}^{-},g_{12}^{+}$, and $g_{12}^{-}$. The distance from point *A* to point *G* is $L_{11}$, and the distance from point *G* to point *R*_1_ is $L_{12}$. The distance from the previous resonator to the next one is L. On the basis of the description in Section 2 about propagation, transmission, and reflection relations, the connections of waves at discontinuities of the LR beam are deduced as follows.

At eight points *R*_1_, *R*_2_, *R*_3_, *R*_4_, *R*_5_, *R*_6_, *R*_7_, and *R*_8_, the equations about attached resonators are shown as Equations (54)–(61), considering the periodic design and modeling analysis can be followed and modified easily.

(54) $$[\begin{array}{cc} A_{p1} & A_{n1}\\ A_{p2} & A_{n2} \end{array}] [\begin{array}{c} {r_{11}}^{+}\\ {r_{11}}^{-} \end{array}]+ [\begin{array}{cc} B_{p1} & B_{n1}\\ B_{p2} & B_{n2} \end{array}] [\begin{array}{c} {r_{12}}^{+}\\ {r_{12}}^{-} \end{array}]=0$$

(55) $$[\begin{array}{cc} A_{p1} & A_{n1}\\ A_{p2} & A_{n2} \end{array}] [\begin{array}{c} {r_{21}}^{+}\\ {r_{21}}^{-} \end{array}]+ [\begin{array}{cc} B_{p1} & B_{n1}\\ B_{p2} & B_{n2} \end{array}] [\begin{array}{c} {r_{22}}^{+}\\ {r_{22}}^{-} \end{array}]=0$$

(56) $$[\begin{array}{cc} A_{p1} & A_{n1}\\ A_{p2} & A_{n2} \end{array}] [\begin{array}{c} {r_{31}}^{+}\\ {r_{31}}^{-} \end{array}]+ [\begin{array}{cc} B_{p1} & B_{n1}\\ B_{p2} & B_{n2} \end{array}] [\begin{array}{c} {r_{32}}^{+}\\ {r_{32}}^{-} \end{array}]=0$$

(57) $$[\begin{array}{cc} A_{p1} & A_{n1}\\ A_{p2} & A_{n2} \end{array}] [\begin{array}{c} {r_{41}}^{+}\\ {r_{41}}^{-} \end{array}]+ [\begin{array}{cc} B_{p1} & B_{n1}\\ B_{p2} & B_{n2} \end{array}] [\begin{array}{c} {r_{42}}^{+}\\ {r_{42}}^{-} \end{array}]=0$$

(58) $$[\begin{array}{cc} A_{p1} & A_{n1}\\ A_{p2} & A_{n2} \end{array}] [\begin{array}{c} {r_{51}}^{+}\\ {r_{51}}^{-} \end{array}]+ [\begin{array}{cc} B_{p1} & B_{n1}\\ B_{p2} & B_{n2} \end{array}] [\begin{array}{c} {r_{52}}^{+}\\ {r_{52}}^{-} \end{array}]=0$$

(59) $$[\begin{array}{cc} A_{p1} & A_{n1}\\ A_{p2} & A_{n2} \end{array}] [\begin{array}{c} {r_{61}}^{+}\\ {r_{61}}^{-} \end{array}]+ [\begin{array}{cc} B_{p1} & B_{n1}\\ B_{p2} & B_{n2} \end{array}] [\begin{array}{c} {r_{62}}^{+}\\ {r_{62}}^{-} \end{array}]=0$$

(60) $$[\begin{array}{cc} A_{p1} & A_{n1}\\ A_{p2} & A_{n2} \end{array}] [\begin{array}{c} {r_{71}}^{+}\\ {r_{71}}^{-} \end{array}]+ [\begin{array}{cc} B_{p1} & B_{n1}\\ B_{p2} & B_{n2} \end{array}] [\begin{array}{c} {r_{72}}^{+}\\ {r_{72}}^{-} \end{array}]=0$$

(61) $$[\begin{array}{cc} A_{p1} & A_{n1}\\ A_{p2} & A_{n2} \end{array}] [\begin{array}{c} {r_{81}}^{+}\\ {r_{81}}^{-} \end{array}]+ [\begin{array}{cc} B_{p1} & B_{n1}\\ B_{p2} & B_{n2} \end{array}] [\begin{array}{c} {r_{82}}^{+}\\ {r_{82}}^{-} \end{array}]=0$$

At free support boundaries *A* and *B*:

(62) $$a^{+}=r_{f}a^{-}$$

(63) $$b^{-}=r_{f}b^{+}$$

Propagation relations are correspondingly derived along *AG*, *GR*_1_, *R*_1_*R*_2_, *R*_2_*R*_3_, *R*_3_*R*_4_, *R*_4_*C*, *CR*_5_, *R*_5_*R*_6_, *R*_6_*R*_7_, *R*_7_*R*_8_, and *R*_8_*B* of the LR frame. In the equations, $f\left(L\right)$ and $f\left(\frac{L}{2}\right)$ represent the propagation matrices along the continuous beam with a distance *L* and $\frac{L}{2}$, respectively.

Along *AG*,

(64) $$g_{11}^{+}=f\left(L_{11}\right)a^{+},\;\;a^{-}=f\left(L_{11}\right)g_{11}^{-}$$

Along *GR*_1_,

(65) $$r_{11}^{+}=f\left(L_{12}\right)g_{12}^{+},\;\;g_{12}^{-}=f\left(L_{12}\right)r_{11}^{-}$$

Along *R*_1_*R*_2_,

(66) $$r_{21}^{+}=f\left(L\right)r_{12}^{+},\;\;r_{12}^{-}=f(L)r_{21}^{-}$$

Along *R*_2_*R*_3_,

(67) $$r_{31}^{+}=f\left(L\right)r_{22}^{+},\;\;r_{22}^{-}=f_{s}(d)r_{31}^{-}$$

Along *R*_3_*R*_4_,

(68) $$r_{41}^{+}=f\left(L\right)r_{32}^{+},\;\;r_{32}^{-}=f(L)r_{41}^{-}$$

Along *R*_4_*C*,

(69) $$c_{1}^{+}=f\left(\frac{L}{2}\right)r_{42}^{+},\;\;r_{42}^{-}=f\left(\frac{L}{2}\right)c_{1}^{-}$$

Along *CR*_5_,

(70) $$r_{51}^{+}=f\left(\frac{L}{2}\right)c_{2}^{+},\;\;c_{2}^{-}=f\left(\frac{L}{2}\right)r_{51}^{-}$$

Along *R*_5_*R*_6_,

(71) $$r_{61}^{+}=f\left(L\right)r_{52}^{+},\;\;\;\;r_{52}^{-}=f\left(L\right)r_{61}^{-}$$

Along *R*_6_*R*_7_,

(72) $$r_{71}^{+}=f\left(L\right)r_{62}^{+},\;\;\;\;r_{62}^{-}=f\left(L\right)r_{71}^{-}$$

Along *R*_7_*R*_8_,

(73) $$r_{81}^{+}=f\left(L\right)r_{72}^{+},\;\;\;\;r_{72}^{-}=f\left(L\right)r_{81}^{-}$$

Along *R*_8_*B*,

(74) $$b^{+}=f\left(L\right)r_{82}^{+},\;\;r_{82}^{-}=f\left(L\right)b^{-}$$

At L joint *C*,

(75) $$c_{1}^{-}=r_{11}c_{1}^{+}+t_{21}c_{2}^{-}$$

(76) $$c_{2}^{+}=r_{22}c_{2}^{-}+t_{12}c_{1}^{+}$$

The relations between the additional force and the generated wave amplitudes are:

(77) $$g_{12}^{+}-g_{11}^{+}=q$$

(78) $$g_{12}^{-}-g_{11}^{-}=-q$$

Combining Equations (54)–(78) and writing them into matrix algebraic form gives:

(79) $$A_{f}z_{f}=F$$

where $A_{f}$ is a 132×132 coefficient matrix, $z_{f}$ is a 132×1 component vector, and F is a 132×1 vector holding the additional transverse forces to the host beam.

The computational method for the FRF is presented below, where the combination of two types of loading (transverse force and bending moment) are considered to the host beam. Assuming the distance from the output point to point *R*_8_ on the LR beam *R*_8_*B* is x, the corresponding deflection can be derived from:

(80) $$y=[1\;1\;1]{f\left(x\right)r}_{82}^{+}+[1\;1\;1]{f\left(-x\right)r}_{82}^{-}$$

Then, the total deflection under two types of applied loadings can be expressed as:

(81) $$y_{t}=\sqrt{y_{f}^{2}+y_{m}^{2}}$$

where $y_{f}$ and $y_{m}$ are respectively defined as the deflection of the output point under transverse force loading and bending moment loading.

From the loading point G, the similar deflection is:

(82) $$x=[1\;1\;1]g_{11}^{+}+[1\;1\;1]g_{11}^{-}$$

Meanwhile, the total deflection of the point that applied two kinds of loadings is:

(83) $$x_{t}=\sqrt{x_{f}^{2}+x_{m}^{2}}$$

where $x_{f}$ and $x_{m}$ are the deflection of the loading point similar to $y_{f}$ and $y_{m}$.

Correspondingly, the computational expression of FRF on the LR beam can be followed by:

(84) $$f=-20lg\frac{y_{t}}{x_{t}}$$

## 4. Numerical Results and Discussion

We combine the relevant conclusions in Section 3 to analyze a specific LR beam structure to verify the proposed analysis method; the systematic process is illustrated below. A finite Timoshenko LR beam with L-joint suspended with eight periodic resonators is applied to verify the accuracy of the proposed approach. The L-joint position is in the middle of the host beam. The physical parameters of the example host beam are as follows: Young’s modulus $E=70\;GN/m^{2}$, Poisson’s ratio v=0.33, shear modulus G=E/2(1+v) related to E and the Poisson’s ratio v, mass density $\rho =2700\;kg/m^{3}$, cross-sectional geometry of the host beam given by $3\times 10^{-3}\times 1\times 10^{-2}m^{2}$, and the shear coefficient is calculated by using κ=10(1+v)/(12+11v). The lattice constant L=0.1 m, the loading site $L_{11}=0.01$ m, $L_{12}=0.09\;m,$ and the distance from the last resonator to the right end of the host beam is also equal to the lattice constant L=0.1 m. The mass and stiffness of the resonators correspondingly are $m_{A}=4.027g$ and $k_{A}=1.6384\times 10^{4}$ N/m, respectively. 

**Figure 11:**
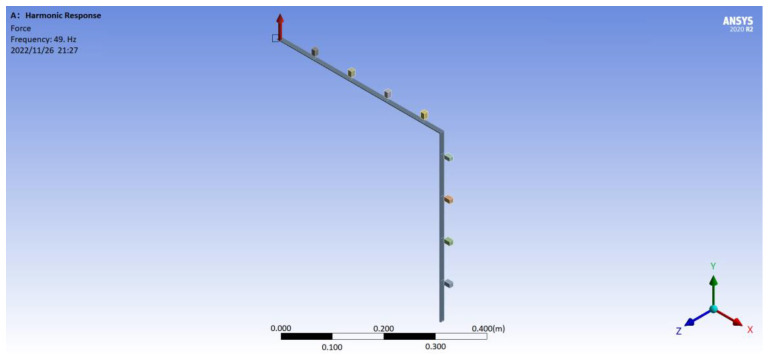
The geometrical model for the simulation.

Figure 11 denotes the geometrical model for the simulation. The simulation analysis was calculated by FEM (ANSYS Workbench software). The mass *m* is realized by the blocks in the model. It is easy to obtain the volume size of the blocks when we choose the same material with the host beam. The spring stiffness is set through the ‘elastic connection’ in the ANSYS Workbench software. With the FEM analysis software, the type of the ‘Analysis Systems’ is ‘Harmonic Response’. In the simulation, element types in the ANSYS model include Solid 186, Solid 187, Conta 174, Combin 14, and Surf 154. The element size is set as beams adopting patch conforming method and mass blocks adopting sweep method. The number of total elements is 1312. With a step of 1 Hz, the harmonic force excitation with band width ranging from 0 Hz to 500 Hz was loaded near the left end of the metamaterial beam in *y* direction when simulating. The metamaterial beam vibrated freely without any boundary limitation at both ends of the beam.

Considering the notable impact of the axial waves on the transverse vibration of L-joint of the LR beam, the longitudinal-force-moment type resonators are applied in the LR beam to further widen the band gaps, where two kinds of band gaps are generated for the different positions of the longitudinal-force-moment type resonators. Firstly, the longitudinal-force-moment type resonators are set at the first two cells of the LR beam (considered as LR beam 2). The other case is that the longitudinal-force-moment type resonators are set at the third and fourth cells of the LR beam (considered as LR beam 3). Similarly, we name the LR beam suspended only with transverse-force type resonators as LR beam 1 here. For LR beam 2, Equations (85) and (86) are as follows; correspondingly, substitute Equations (54) and (55) to form the coefficient matrix. For LR beam 3, Equations (56) and (57) are replaced with Equations (87) and (88) as follows:

(85) $$[\begin{array}{cc} C_{p1} & C_{n1}\\ C_{p2} & C_{n2} \end{array}] [\begin{array}{c} {r_{11}}^{+}\\ {r_{11}}^{-} \end{array}]+ [\begin{array}{cc} D_{p1} & D_{n1}\\ D_{p2} & D_{n2} \end{array}] [\begin{array}{c} {r_{12}}^{+}\\ {r_{12}}^{-} \end{array}]=0$$

(86) $$[\begin{array}{cc} C_{p1} & C_{n1}\\ C_{p2} & C_{n2} \end{array}] [\begin{array}{c} {r_{21}}^{+}\\ {r_{21}}^{-} \end{array}]+ [\begin{array}{cc} D_{p1} & D_{n1}\\ D_{p2} & D_{n2} \end{array}] [\begin{array}{c} {r_{22}}^{+}\\ {r_{22}}^{-} \end{array}]=0$$

(87) $$[\begin{array}{cc} C_{p1} & C_{n1}\\ C_{p2} & C_{n2} \end{array}] [\begin{array}{c} {r_{31}}^{+}\\ {r_{31}}^{-} \end{array}]+ [\begin{array}{cc} D_{p1} & D_{n1}\\ D_{p2} & D_{n2} \end{array}] [\begin{array}{c} {r_{32}}^{+}\\ {r_{32}}^{-} \end{array}]=0$$

(88) $$[\begin{array}{cc} C_{p1} & C_{n1}\\ C_{p2} & C_{n2} \end{array}] [\begin{array}{c} {r_{41}}^{+}\\ {r_{41}}^{-} \end{array}]+ [\begin{array}{cc} D_{p1} & D_{n1}\\ D_{p2} & D_{n2} \end{array}] [\begin{array}{c} {r_{42}}^{+}\\ {r_{42}}^{-} \end{array}]=0$$

where the coefficient matrices $C_{p1}$, $C_{p2}$, $C_{n1},C_{n2}$, $D_{p1}$, $D_{p2}$, $D_{n1}$, and $D_{n2}$ in Equations (85)–(88) can be found in Section 2.7.

**Figure 12:**
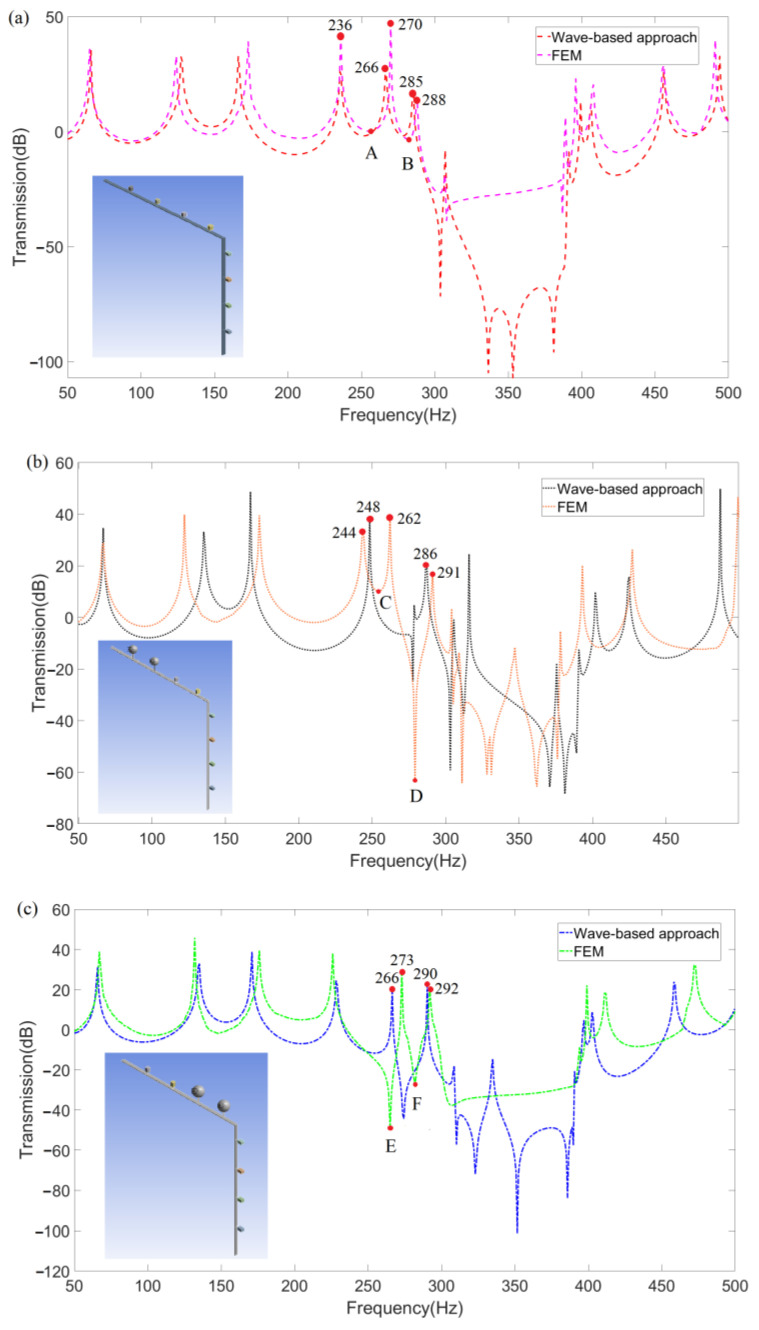
The FRFs of the LR beam using the wave-based method and FEM (**a**) suspended with transverse-force type resonators, (**b**) suspended with longitudinal-force-moment type resonators set at the first two cells, (**c**) suspended with longitudinal-force-moment type resonators set at the third and fourth cells.

Then, Figure 12a–c represent the comparison of frequency response function (FRF) curves of three kinds of LR beams obtained using both wave-based vibration approach and FEM. The corresponding models of each LR beam are also inserted in each figure. Figure 12a–c show the FRFs of LR beam 1, 2, and 3, respectively, where the wave-based approach is further verified as more accurate and easier to modify models than FEM needing to model repeatedly. Moreover, the wave-based approach is also convenient for subsequent analysis and design.

**Figure 13:**
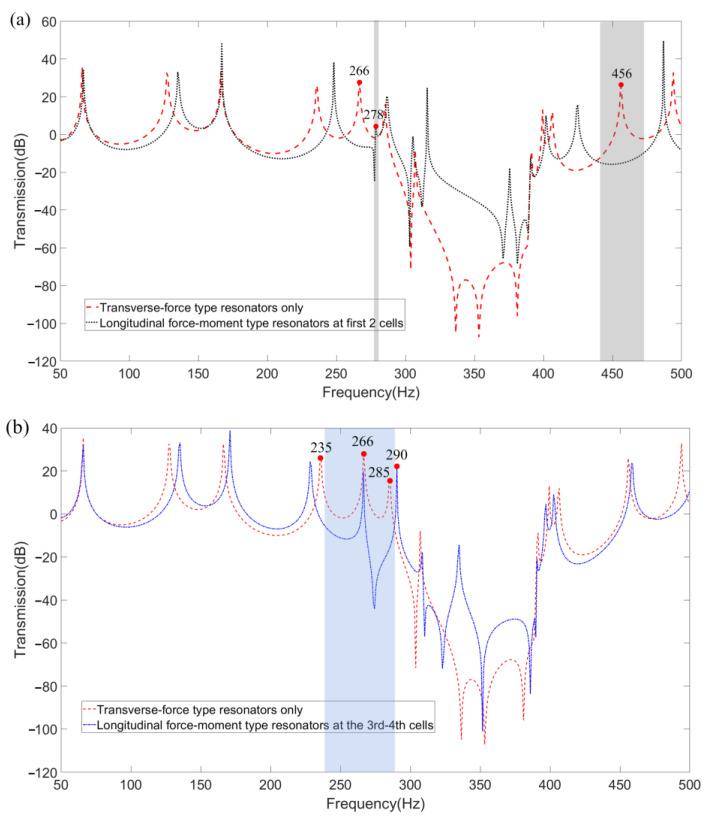
The comparison FRFs of the LR beam (**a**) suspended with transverse-force type resonators only and longitudinal-force-moment type resonators at the first two cells, (**b**) suspended with transverse-force type resonators only and longitudinal-force-moment type resonators at the 3rd−4th cells.

To represent the widening effect on the band gaps more clearly, the FRF results obtained by wave-based vibration approach in Figure 12a–c are further integrated into Figure 13a,b, where the band gaps widened by substituting the first two and 3rd–4th cells of the transverse-force type resonators on the LR beam into longitudinal-force-moment type resonators are, respectively, shown as gray in Figure 13a and blue in Figure 13b. Considering the vibration loss of the LR beam, the band-gap is defined as the FRF curve below −5 dB. The value of natural frequencies at peak points around the widened band-gap are marked in Figure 13. Preliminary analysis of the band-gap widen effect is that the longitudinal-force-moment type resonators have better control on the transmission of axial waves, which may cause both transverse and longitudinal vibrations of the LR beam with L-joint. Furthermore, as shown in Figure 13b, the widening effect on the low-frequency band-gaps is more outstanding when the longitudinal-force-moment type resonators are set at the third and fourth cells. 

**Figure 14:**
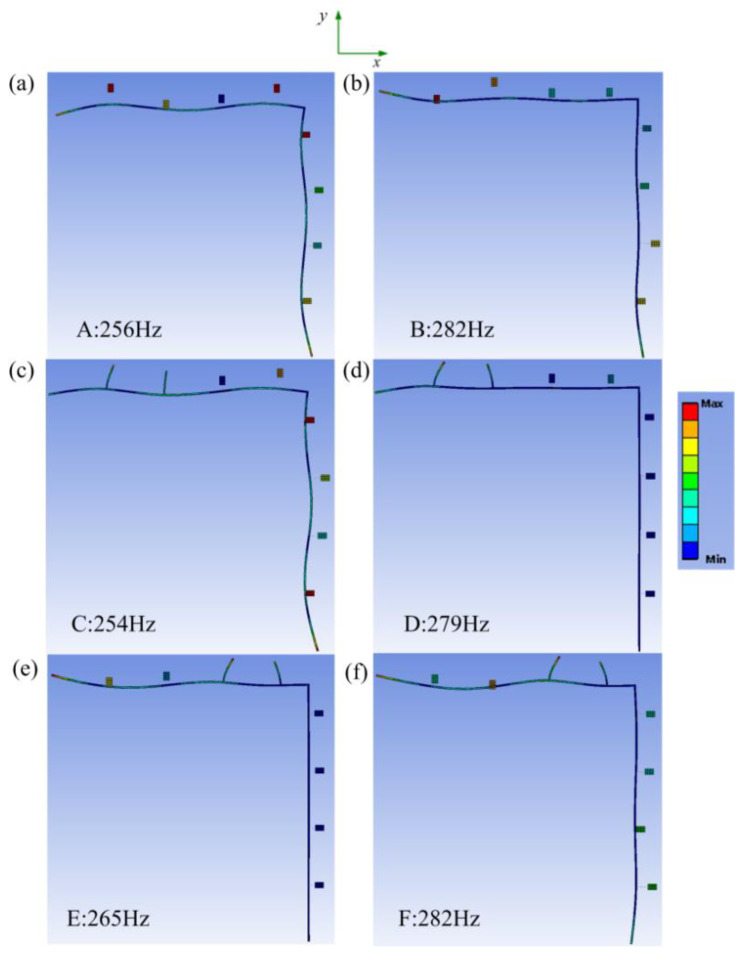
The analysis of steady-state vibration deformations of (**a**) point A; (**b**) point B; (**c**) point C; (**d**) point D; (**e**) point E; (**f**) point F.

For a better physical understanding of the broadband effect of the LR beam 2 and LR beam 3, the steady-state vibration deformations of six specific points A, B, C, D, E, and F marked in Figure 12a–c are further analyzed and shown in Figure 14a–e. Here, points A, C, and E, respectively, are corresponding to the trough near 260 Hz of transmission curves for three kinds of LR beams. Meanwhile, points B, D, and F, respectively, are corresponding to the trough near 280 Hz. The purpose of choosing these points is to show the results of the comparison of the band gap clearly widened, where points A and C are corresponding to point E, and point B is corresponding to points D and F. The value of natural frequencies around the specific choosing points are marked in Figure 12 as well. As shown in Figure 14a,c,e, the attenuation effect of the LR beam with longitudinal-force-moment type resonators set at the first two cells is not observable with the incident wave near 260 Hz. The attenuation effect of the LR beam with longitudinal-force-moment type resonators set at the 3rd–4th cells is obviously more remarkable. It is probably because the longitudinal-force-moment type resonators set at the first two cells cannot restrain the propagation of axial waves well. By comparing the deformations in Figure 14b,d,f, the vibration suppression of LR beam 2 and 3 are both obvious when the vibration is near 280 Hz. Especially, LR beam 2 (when the longitudinal-force-moment type resonators are set at the first two cells) can relatively suppress vibration better. 

In summary, the method used in experimental investigations and for the analysis of experimental results has been further introduced in this part. For low-frequency vibration, the LR beam with longitudinal-force-moment type resonators set at the 3rd–4th cells can broaden the band gaps more effectively. 

## 5. Concluding Remarks

In this paper, the wave-based approach further developed was to study the transmission characteristics of the LR beam with L-joint, which is common in engineering practices (band-gap behavior, propagation characteristics, dispersion properties, etc.). Based on the proposed approach, the resonator was considered for applying force and generating waves into the host beam. At the resonator’s attached points, the reflection and transmission matrices were deduced. Additionally, the propagation characteristic of a finite LR beam with L-joint suspended with eight transverse-force type resonators was analyzed. By simply combining the involved reflection and transmission matrices, the vibration analysis procedure can be obtained, where more details have been introduced by Leamy [42]. The high efficiency and accuracy of the proposed approach for analyzing the finite LR beams with L-joint has also been verified by comparing the analysis results with FEM in Section 4. Considering the effect of an axial wave on the vibration of the LR beam with L-joint, the band gaps of another two kinds of LR beam with longitudinal-force-moment type resonators at the first two cells and the 3rd–4th cells were further analyzed to verify the widened effect on the band gaps. By analyzing the steady-state vibration deformations of the six specific points of three kinds of the LR beams, we found that longitudinal-force-moment type resonators at the 3rd–4th cells can better suppress the propagation of the axial waves in the LR beam in low-frequency range. Therefore, the band gap of the LR beam at low-frequency range can be broadened more faithfully. In this paper, the wave-propagation method is applied to LR beams with L-joint, mainly due to its accuracy, good applicability, and repeatability of procedures compared to other approaches. 

Generally, this paper provides direction in the design and analysis of the common LR beam (with L-joint) in engineering practices for low-frequency vibration attenuation. The results are specific to the structure presented, but they have the role of guiding the design and analysis of vibration damping structure in practical engineering. In addition, the proposed analysis can further simplify future work such as structural health monitoring and damage detection. Applying the proposed approach to study the band-gap properties of space LR frame is an interesting topic in our future investigations.

## Figures and Tables

**Table 1:** Notations and definitions.

Parameter	Value
E	Young’s modulus
v	Poisson’s ratio
G	Shear modulus
ρ	Mass density
*A*	Area of the cross section
κ	Shear coefficient
k	Spring stiffness
*L*	Lattice constant
ω	Frequency
*m*	Resonator mass
ψ	Rotation angle
I	Area moment of inertia
w	Transverse deflection
u	Longitudinal deflection

**Table 2:** List of key acronyms used in this paper.

Parameter	Value
LR	Locally resonant
AMs	Acoustics metamaterials
EMs	Elastic metamaterials
PCs	Phononic crystals
SEM	Spectral element method
TMM	Transfer matrix method
FEM	Finite element method
FRF	Frequency response function

## Data Availability

Not applicable.
